# ProkBERT PhaStyle: accurate phage lifestyle prediction with pretrained genomic language models

**DOI:** 10.1093/bioadv/vbaf188

**Published:** 2025-11-09

**Authors:** Judit Juhász, Noémi Ligeti-Nagy, Babett Bodnár, János Juhász, Sándor Pongor, Balázs Ligeti

**Affiliations:** Faculty of Information Technology and Bionics, Pázmány Péter Catholic University, Budapest 1083, Hungary; Language Technology Research Group, ELTE Research Centre for Linguistics, Budapest 1068, Hungary; Faculty of Information Technology and Bionics, Pázmány Péter Catholic University, Budapest 1083, Hungary; Faculty of Information Technology and Bionics, Pázmány Péter Catholic University, Budapest 1083, Hungary; Institute of Medical Microbiology, Semmelweis University, Budapest 1089, Hungary; Faculty of Information Technology and Bionics, Pázmány Péter Catholic University, Budapest 1083, Hungary; Faculty of Information Technology and Bionics, Pázmány Péter Catholic University, Budapest 1083, Hungary

## Abstract

**Motivation:**

Phage lifestyle prediction, i.e. classifying phage sequences as virulent or temperate, is crucial in biomedical and ecological applications. Phage sequences from metagenome or virome assemblies are often fragmented, and the diversity of environmental phages is not well known. Current computational approaches often rely on database comparisons that require significant effort and expertise to update. We propose using genomic language models (LMs) for phage lifestyle classification, allowing efficient direct analysis from nucleotide sequences without the need for sophisticated preprocessing pipelines or manually curated databases. We trained three genomic LMs (DNABERT-2, Nucleotide Transformer, and ProkBERT) on datasets of short, fragmented sequences. These models were then compared with dedicated phage lifestyle prediction methods in terms of accuracy, prediction speed, and generalization capability.

**Results:**

ProkBERT PhaStyle achieves accuracy comparable to, and in many cases higher than, state-of-the-art models across various scenarios. It demonstrates the ability to generalize to unseen data in our benchmarks, accurately classifies phages from extreme environments, and also demonstrates high inference speed.

**Availability and implementation:**

Genomic LMs offer a simple and computationally efficient alternative for solving complex classification tasks, such as phage lifestyle prediction. ProkBERT PhaStyle’s simplicity, speed, and performance suggest its utility in various ecological and clinical applications.

## 1 Introduction

Bacteriophages, the viruses targeting bacteria, exhibit distinct lifestyles: virulent phages destroy host bacteria through the lytic cycle, potentially clearing pathogenic bacterial infections quickly; temperate phages, on the other hand, can use both the lytic and lysogenic cycles, meaning they can integrate into the bacterial genome and can transfer genes, influencing bacterial evolution and pathology (Howard-Varona *et al.* 2017). Understanding phage-host interactions is pivotal for advancing medical and environmental biotechnology (Cao *et al.* 2022, Mirzaei and Deng 2022), such as phage therapeutic applications (Gutiérrez and Domingo-Calap 2020, Cao *et al.* 2022, Strathdee *et al.* 2023) and microbiome engineering (Foo *et al.* 2017, Bhattarai and Kashyap 2020).

Despite the critical role of phages, predicting their phenotypes, particularly their lifestyles, remains less explored. Existing tools for phage lifestyle prediction can be categorized into nucleotide-based and protein-based approaches. Temperate and virulent phages possess distinct characteristics: temperate phages often encode toxins, integrase, and excisionase genes, whereas virulent phages typically encode lysis and nucleotide metabolism-related genes (Shkoporov *et al.* 2019, Moura de Sousa *et al.* 2021). These features form the basis for heuristic-driven phage lifestyle predictors. Protein-based tools, such as PHACTS, utilize protein information and machine learning techniques like Random Forests to predict phage lifestyles (McNair *et al.* 2012). BACPHLIP (Hockenberry and Wilke 2021), which uses profile hidden Markov models (HMMs) to identify lysogeny-associated protein domains, and PhaTYP (Shang *et al.* 2023), which relies on database searches to construct protein sentences for prediction, are examples of this approach. Conversely, nucleotide-based tools like PhagePred utilize a k-mer frequency-based Markov model to calculate the similarity of query contigs to known temperate and virulent phages (Song 2020). DeePL chunks the DNA sequence into 100 bp length fragments, identifies the lysogenic genes followed by subsequent aggregation and thresholding of prediction scores step (Zhang *et al.* 2024). DeePhage, another alignment-free approach, employs a convolutional neural network to learn local patterns, enabling it to classify contigs effectively (Wu *et al.* 2021).

Several challenges persist in phage lifestyle prediction: (i) accurately labeling fragmented phage sequences, (ii) achieving computational efficiency, and (iii) recognizing and classifying phages that are underrepresented or absent in the training set. Phage sequences derived from viromic and metagenomic studies are often incomplete and fragmented, reducing the accuracy of current methods such as BACPHLIP, PHACTS, and PhagePred for shorter fragments (<5 kb) (Song 2020, Wu *et al.* 2021). Despite advancements in viral catalogs (Paez-Espino *et al.* 2016, Camarillo-Guerrero *et al.* 2021, Nayfach *et al.* 2021b, Camargo *et al.* 2023), the human virome remains under-characterized (Shah *et al.* 2023). This leads to biases and computational expenses in database-based approaches, such as PhaTYP and BACPHLIP, which often fail in “out-of-sample” scenarios, where they encounter previously unseen phages (Hockenberry and Wilke 2021). Addressing these challenges is essential for developing robust phage lifestyle prediction models.

Transformer-based language models (LMs) have demonstrated excellent generalization capabilities in natural language processing tasks (Vaswani *et al.* 2017), making them good candidates for data-scarce scenarios common in biomedical fields. In biological sequences, transformer models, such as MSA Transformer (Rao *et al.* 2021), ESM (Lin *et al.* 2023, Rives *et al.* 2021), AlphaFold2 (Jumper *et al.* 2021), and openFold (Ahdritz *et al.* 2024), have been effectively applied. For nucleotide sequences, models, such as DNABERT (Ji *et al.* 2021, Zhou *et al.* 2023), GENA-LM (Fishman *et al.* 2023), Nucleotide Transformer (NT) (Lopez *et al.* 2025), Mamba (Gu and Dao 2023), HyenaDNA (Nguyen *et al.* 2023), and ProkBERT, a model pretrained on microbial sequences (Ligeti *et al.* 2023) show promise, but they have not yet been employed for phage lifestyle prediction tasks.

In this work, we fine-tune universal genomic LMs (NT: 50–500 m parameters, DNABERT-2: 117 m, and ProkBERT: 21–26 m parameters) and then benchmark them against the current state of the art phage lifestyle prediction tools (BACPHLIP, DeePhage, PhaTYP). The benchmarking focuses on three aspects, (i) how well the pretrained LMs can classify low quality and fragmented viral contigs, (ii) the prediction speed of each method, and (iii) how well the models perform on “unseen,” out-of-the-sample predictions.

We demonstrate that the task of phage lifestyle prediction can be solved efficiently and with high classification accuracy directly from nucleotide sequences using a simple pipeline. This approach is fast, requires no arbitrary thresholding, and provides improved generalization by leveraging the advantages of large genomic LM-derived sequence representations. Our results show that ProkBERT PhaStyle performed robustly in scenarios involving unseen phages and fragmented sequences, suggesting its potential as a useful tool for phage lifestyle prediction in diverse ecological and clinical settings.

## 2 Methods

The efficacy and applicability of machine learning algorithms are profoundly impacted by the quality of the training dataset and the comprehensiveness of the benchmarking process. A recurrent challenge in bioinformatics is the application of developed computational tools to sequences that were not represented or were underrepresented in the model’s training data, potentially leading to biased predictions. To address this issue and simulate real-world application scenarios, our study specifically focuses on testing models against “unseen” genera, with an emphasis on *Escherichia* and extremophile groups. In this study, we used five independent data sources (for an overview of each dataset, see Table 1, available as supplementary data at *Bioinformatics Advances* online). Test datasets comprise experimentally confirmed novel virulent phages sourced from various environments, as detailed in subsequent sections. Moreover, to prevent overlap between training and test sets, any phage sequence in the training data exhibiting an average nucleotide identity (ANI) ≥80% with a sequence in the test dataset was excluded. We estimate and report performance across diverse fragment lengths (500, 2000, 10 000 bp; see Table 2, available as supplementary data at *Bioinformatics Advances* online for an overview), as such contigs are frequently disregarded or eliminated from further analytical steps due to their incompleteness. The quality of the contigs was assessed using checkV (Nayfach *et al.* 2021a), and while both the training and test sequences are high quality or complete (98%), the simulated contigs are low quality (99.97%) as demonstrated in Tables 3 and 4, available as supplementary data at *Bioinformatics Advances* online.

### 2.1 Datasets

#### 2.1.1 Training and validation datasets

The training and validation datasets were assembled to ensure high-quality annotations and a broad representation of phage lifestyles, building on the work of Mavrich and Hatfull (2017) and the BACPHLIP study by Hockenberry and Wilke (Mavrich and Hatfull 2017, Hockenberry and Wilke 2021). We compiled two training datasets: one in which *Escherichia*-infecting phages were excluded based on ANI and host infection annotation (Strict-Holdout with ANI ≥80% filter), and one without such filtering (Standard-Holdout, BACPHLIP only). These datasets include 2114 sequences: 1798 in the training set (non-*Escherichia* phages) and 316 in the validation set (*Escherichia* phages).

#### 2.1.2 Test dataset 1—BASEL collection

The BASEL (BActeriophage SElection for your Laboratory) collection comprises 105 *Escherichia coli* phage isolates (including their reverse complements), assembled to capture maximal taxonomic and functional diversity while remaining experimentally tractable on a single host background (Humolli *et al.* 2025). All isolates were obtained from environmental samples (e.g. wastewater, river water, park ponds, bird baths) by direct plaque assays on *E.coli* K‐12 strains. This collection provides a well-characterized, experimentally validated panel of *E.coli* phages spanning ∼30 genera, for systematic studies of phage–host interactions. As a control, 100 temperate phages were selected from the TemPhD database (Zhang *et al.* 2022), identified based on *Escherichia* sequence data.

#### 2.1.3 Test dataset 2—Extremophile collection

This test dataset is a curated collection of bacteriophages isolated from extreme environments (see Table 5, available as supplementary data at *Bioinformatics Advances* online)—deep-sea (Mariana Trench), acidic, and arsenic-rich environments. The virulent phages live in arsenic-rich microbial mats in cold climates (Bujak *et al.* 2022). The temperate phages infect the psychrotolerant deep-sea bacterium *Aurantimonas* and *Halomonas* (Engelhardt *et al.* 2013, Yoshida *et al.* 2015, Su *et al.* 2023). The collection also contains a bacteriophage with hosts like *Acidithiobacillus caldus*, an extremophilic bacterium thriving in highly acidic environments (pH < 2) (Tapia *et al.* 2012).

#### 2.1.4 Test dataset 3—Guelin (*Escherichia*) collection

A collection of 96 taxonomically diverse *Escherichia* bacteriophages (Guelin collection) was used for testing the methods. This collection was constructed by Gaborieau *et al.* (2023) for experimentally studying virulent host-phage interactions in different phage concentrations. It contains the archetypical T4 and T7 phages of *E.coli* and 94 other phages isolated from different wastewater treatment plants around Paris (France) over 10 years. Their lytic capabilities were experimentally evaluated against different members of the *Escherichia* genus. The collection contains unique members with <98% nucleotide identity or different host-range. As a control, 100 temperate phages were selected from the TemPhD database.

### 2.2 Application of current phage lifestyle prediction methods

DeePhage employs one-hot encoding for vectorizing individual sequences. The implementation relies on Keras and MATLAB; therefore, we encapsulated DeePhage into both Docker and Apptainer containers. DeePhage was used with the default settings and GPU support. All datasets were evaluated at both the sequence and segment levels.

PhaTYP uses a BERT architecture for classification, but it operates on protein sentences, rather than nucleotide or amino-acid sequences. It was used with multithread support and was encapsulated into an Apptainer. The segment datasets were evaluated separately from the complete sequences.

BACPHLIP was installed via pip (version 0.9.6). The “multi_fasta” option was used for evaluating the sequence datasets. However, the segment datasets were not evaluated, as this is against the recommended usage by the authors.

All Dockerfiles, Singularity definition files are available on the GitHub page. The Docker containers are also available on Docker Hub, and the Apptainer images are shared on Zenodo.

### 2.3 Running time and inference speed estimation

The running time was measured on a set of 1000 randomly selected sequences from the BACPHLIP dataset. We evaluated methods that support GPU inference (genomic LMs, PhaTYP, DeePhage), with each model allocated one NVIDIA Tesla A100 GPU, 8 CPU cores, and 32 GB of RAM. The batch size for evaluation was adjusted based on the input sequence length and model size, as described previously. The total elapsed time accounted for all processing stages, including initialization, model and dataset loading, parsing, preprocessing of the sequence datasets (such as segmentation and tokenization), inference, and report generation (including weighted voting). Each measurement was independently repeated three times under identical conditions, and the average running time was reported. The inference speed is defined as the rate of classification (number of classified nucleotides per second).

### 2.4 Evaluation metrics

We used standard metrics to evaluate binary classification performance, including balanced accuracy, accuracy, sensitivity, specificity, F1 score, and Matthews Correlation Coefficient (MCC). The cross-entropy loss function was used during the training of the genomic LM. Formal definitions of these metrics can be found in the Supplementary Material (section: Evaluation metrics and definitions), available as supplementary data at *Bioinformatics Advances* online.

### 2.5 Data preprocessing

The results of metagenome and virome assemblies are frequently fragmented, with the majority of identified contigs being incomplete, typically ≤10 kb (Ho *et al.* 2023). To simulate these cases, we constructed sets of short fragments L=[500,2000,10000 bp] by random sampling from the phage contigs of the test datasets (BASEL, Extremophile, and Guelin collections with an equal number of virulent and temperate contigs). Next, we assessed the quality of both the training and test set results. The training dataset was segmented with an expected coverage of 10× and a segment length of 512 bp, as described in Ligeti *et al.* (2023). In contrast, a contiguous segmentation strategy was applied to the test set due to its practicality.

In most practical genome assemblies, strand orientation is unknown; thus, we included reverse complement sequences for all genomic data.

#### 2.5.1 Ensuring dataset independence through similarity estimation

To avoid data leakage, we employed two different approaches. First, we implemented a sketching-based solution for estimating the similarity between phages using the MinHash algorithm, as described in the Supplementary Text, available as supplementary data at *Bioinformatics Advances* online. Briefly, a signature containing 300 elements was generated from nucleotide k-mers of length 21, using the MurmurHash3 hash function with a seed value of 3. The Jaccard coefficient was then computed for each potential pair of sequences based on their respective signatures. Second, we applied FastANI (Jain *et al.* 2018) in an all-against-all fashion to identify similar sequences in the datasets and detect close relatives. Any sequence in the training data showing at least 80% ANI over 80% of its length with any genome in the test set was removed. While this threshold (80%) (usually 95% is used for species-level classification) excludes closely related organisms, it is not designed to exclude more distantly related ones.

### 2.6 Phage lifestyle prediction with genomic language models

Phage lifestyle prediction is formulated as a binary classification problem, similarly to the approaches of BACPHLIP, DeePhage, and PhaTYP. The task is to distinguish between virulent and non-virulent phage sequences, with temperate phages considered as the negative class.

The models used in this study, DNABERT-2, NT, and ProkBERT, are all encoder architectures. These models take tokenized sequences as input and produce vector representations as output. Classification is performed based on these output vectors. Training is conducted under a transfer learning paradigm; which is carried out as follows: a new model with a binary classification head is initialized from the pretrained model weights, and then the model weights are updated at each training iteration step to recognize virulent phage sequences. The models differ in various aspects such as size, tokenization strategy, and pretraining datasets.

#### 2.6.1 ProkBERT PhaStyle

**Figure 1.**
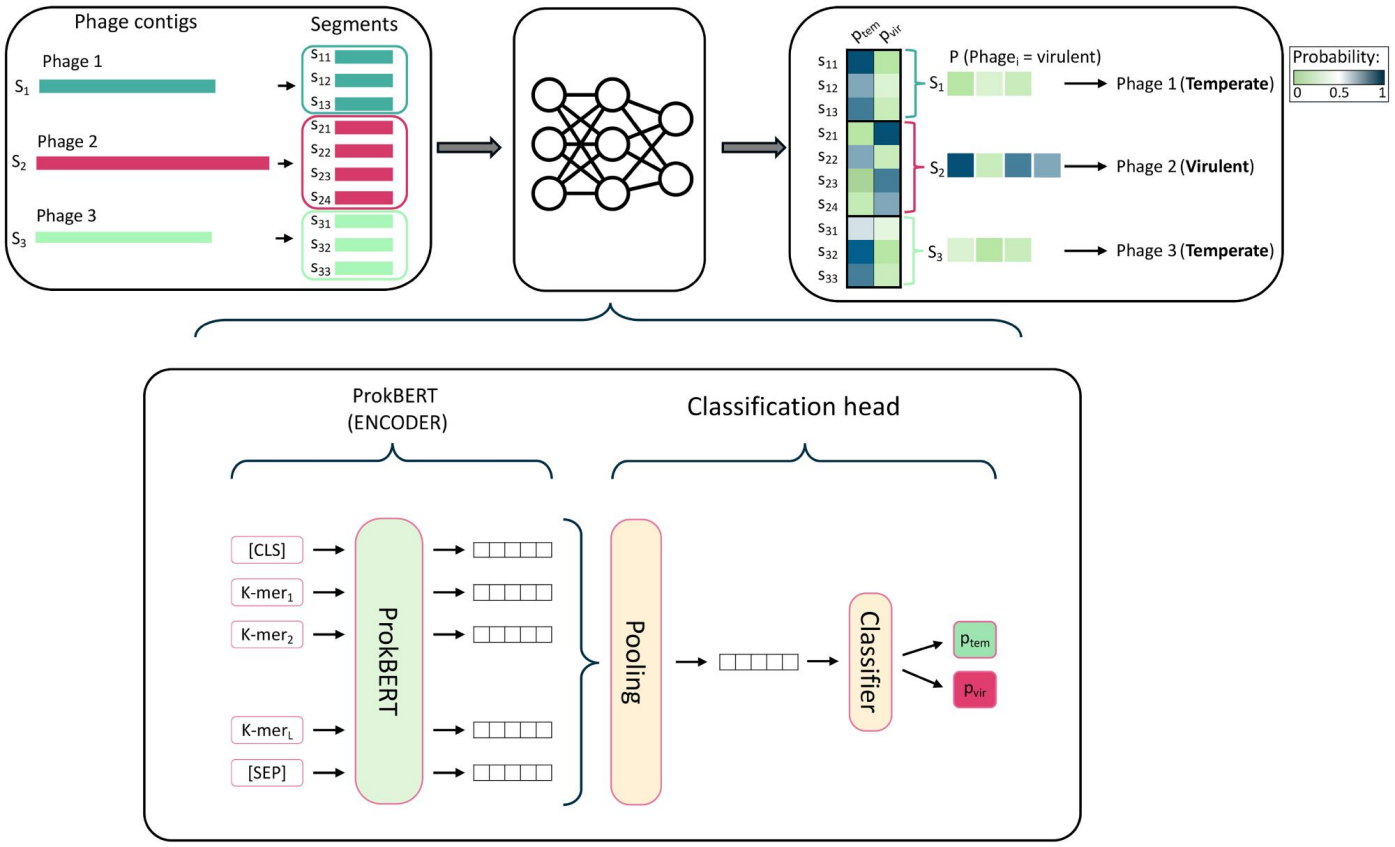
Methodology for phage lifestyle prediction using ProkBERT. The diagram illustrates the process of predicting phage lifestyles from nucleotide sequences. Phage contigs (S_1_, S_2_, S_3_) are segmented into smaller sequences (e.g. S_11_, S_12_, S_13_), which then are inputs to the ProkBERT encoder. The encoder processes these segments using k-mer tokenization and special tokens ([CLS], [SEP]) to generate hidden states. A classification head pools these representations to predict the probabilities of each segment being virulent (P_vir_) or temperate (P_tem_). Then the likelihood of each segment’s lifestyle is calculated. Finally, these probabilities are aggregated to classify the entire phage contig as either virulent or temperate.

The ProkBERT model family (mini, mini-long, and mini-c) accommodates different sequence vectorizations, tokenized with Local Context Aware (LCA) k-mers for efficient representation. Tokenization granularity varies across models, with mini and mini-long using 6-mers and mini-c relying on character-level tokens (see Fig. 1 for an overview of the methodology).

Each model contains 2˜0M parameters, 6 layers, and 6 attention heads. The encoder generates token representations, which are pooled with learnable weights for classification (illustrated in Fig. 1). For detailed architecture and classification steps, see Supplementary Materials, available as supplementary data at *Bioinformatics Advances* online.

The models were fine-tuned on the Komondor HPC system with AdamW optimization and adjusted batch size.

##### 2.6.1.1 Weighted voting for contig classification

To classify entire phage contigs, predictions from individual segments are combined using a weighted voting method, producing a single probability score per contig. See the Supplementary Material (Section 1.1), available as supplementary data at *Bioinformatics Advances* online for the formalized approach.

#### 2.6.2 Other genomic language models

DNABERT-2, a Transformer Encoder with 117M parameters, optimized for genome analysis, uses SentencePiece and BPE for DNA tokenization and incorporates ALiBi and Flash Attention for performance enhancement (Press *et al.* 2021, Dao *et al.* 2022). Pretrained on genomes from 135 species (32.49 billion bases), it was fine-tuned for phage lifestyle prediction using the “AutoModelForSequenceClassification” class, batch sizes of 196 (512 bp) and a learning rate of 0.0001 with AdamW optimizer.

The NT was pretrained on the human reference genome and 850 additional genomes (Lopez *et al.* 2025). In this application, we used the updated models of the smallest and largest sizes (50M and 500M parameters). The models use a 6-mer tokenization scheme with character-level adjustments. Fine-tuning used batch sizes adjusted to input and model sizes and a learning rate of 0.0001, with other parameters matching those for ProkBERT.

## 3 Results

### 3.1 Benchmarking

The performance of the new LM model-based approaches and current state-of-the-art methods was compared on phage lifestyle datasets: (i) the BASEL collection, (ii) phages from extreme environments, and (iii) *Escherichia* phages. The evaluation was conducted on various fragment lengths to simulate real-world applications where viral sequences are often fragmented. To measure the out-of-sample scenario, two models were trained: one in which *Escherichia*-infecting phages and their relatives (quantified by sequence similarity) were excluded (Strict-Holdout), and one without such exclusion (Standard-Holdout). In the first case, the gLMs are at a disadvantage compared to PhaTYP and DeePhage, since the latter’s training data include *Escherichia*-infecting phages. The second case (Standard-Holdout) represents applying models to new phage isolates that may be novel species or variants but whose close relatives are covered by the initial training dataset. In addition, we analyze the inference speeds of the different approaches.

#### 3.1.1 Performance on the BASEL collection

The BASEL collection (Humolli *et al.* 2025) comprises experimentally validated *E.coli* phage genomes spanning ∼30 genera. Table 1 summarizes accuracy, MCC, sensitivity, and specificity under two training-validation schemes. At 500 bp, ProkBERT mini and ProkBERT mini‐long both achieved balanced accuracy 0.88−0.93 and MCC 0.75−0.86, outperforming DNABERT‐2 (accuracy: 0.83–0.91, MCC: 0.67–0.82), NT50/NT500 (accuracy: 0.81–0.92, MCC: 0.62–0.85), DeePhage (accuracy: 0.84), and PhaTYP (accuracy: 0.87). In the *Strict‐Holdout* setting, ProkBERT variants yielded sensitivity up to 0.91 and specificity up to 0.87; under *Standard‐Holdout*, they reached sensitivity up to 0.97 and specificity up to 0.89. Similar trends were observed at 2000 and 10 000 bp fragment lengths. Generally, the models perform better when larger contextual genomic information is available, though the performance gain varies by method. Interestingly, even under the more stringent *Strict‐Holdout* exclusion, the ProkBERT models outperformed DeePhage and PhaTYP. When the *E.coli* sequences were included in the training data, the advantage of the gLM-based models became more pronounced. All gLMs outperformed PhaTYP and DeePhage at all fragment lengths.

**Table 1.** Performance comparison of models on the BASEL collection test set across various segment lengths.^a^

Model	Strict-Holdout (ANI ≧ 80 %)				Standard-Holdout			
	Acc.	MCC	Sens.	Spec.	Acc.	MCC	Sens.	Spec.
500 bp								
ProkBERT-mini	**0.88**	**0.75**	0.88	0.87	**0.93**	**0.86**	**0.97**	**0.89**
ProkBERT-mini-long	**0.88**	**0.75**	**0.91**	0.84	**0.93**	**0.86**	**0.97**	**0.89**
DNABERT-2	0.83	0.67	0.89	0.77	0.91	0.82	**0.97**	0.84
NT500	0.83	0.67	0.86	0.81	0.92	0.85	0.96	0.88
NT50	0.81	0.62	0.84	0.77	0.88	0.76	0.93	0.83
DeePhage	0.84	0.68	0.79	**0.88**	0.84	0.68	0.79	0.88
PhaTYP	0.87	0.74	0.86	**0.88**	0.87	0.74	0.86	0.88
2000 bp								
ProkBERT-mini	**0.95**	**0.89**	0.95	**0.95**	**0.97**	**0.95**	**0.99**	**0.96**
ProkBERT-mini-long	**0.95**	**0.89**	**0.96**	0.93	**0.97**	**0.95**	**0.99**	**0.96**
DNABERT-2	0.9	0.81	0.94	0.86	0.95	0.91	**0.99**	0.92
NT500	0.92	0.83	0.92	0.91	**0.97**	0.94	**0.99**	0.95
NT50	0.89	0.79	0.92	0.87	0.94	0.88	0.97	0.91
DeePhage	0.92	0.84	0.87	0.96	0.92	0.84	0.87	**0.96**
PhaTYP	0.88	0.76	0.9	0.86	0.88	0.76	0.9	0.86
10 000 bp								
ProkBERT-mini	0.96	0.93	0.95	0.97	**0.99**	**0.98**	**0.99**	**0.99**
ProkBERT-mini-long	**0.97**	**0.94**	**0.97**	0.97	**0.99**	**0.98**	**0.99**	**0.99**
DNABERT-2	0.94	0.88	0.95	0.92	0.98	0.97	**0.99**	0.97
NT500	0.95	0.9	0.94	0.96	**0.99**	0.97	**0.99**	0.98
NT50	0.93	0.86	0.92	0.94	0.97	0.94	0.98	0.96
DeePhage	0.94	0.89	0.89	**0.99**	0.94	0.89	0.89	**0.99**
PhaTYP	0.89	0.78	0.9	0.87	0.89	0.78	0.9	0.87

a Best performances in each segment are highlighted in bold. The metrics in the table are as follows: Acc.: accuracy, MCC: Matthews’ Correlation Coefficient, Sens.: sensitivity, and Spec.: specificity. Strict-Holdout refers to the setting where *Escherichia*-infecting phages and their relatives (quantified by sequence similarity) were excluded from the training data; Standard-Holdout shows where not.

#### 3.1.2 Performance on extremophile dataset

Phages isolated from extreme environments—such as deep‐sea trenches, high‐pressure zones, cold ecosystems, and highly acidic settings—are comparatively underrepresented in existing reference collections (Ren *et al.* 2020). As a result, they provide a rigorous test of a model’s ability to generalize beyond well‐characterized viral groups. Table 2 summarizes the evaluation of several approaches on simulated fragments (500, 2000, and 10 000 bp) from this Extremophile collection.

**Table 2.** Performance comparison of models on the Extremophile test set across various segment lengths.^a^

Model	Strict-Holdout (ANI ≧ 80 %)				Standard-Holdout			
	Acc.	MCC	Sens.	Spec.	Acc.	MCC	Sens.	Spec.
500 bp								
ProkBERT-mini	0.89	0.8	**0.99**	0.8	**0.91**	**0.84**	**1**	0.83
ProkBERT-mini-long	**0.9**	**0.82**	**0.99**	0.81	**0.91**	**0.84**	0.99	0.84
DNABERT-2	0.87	0.77	**0.99**	0.76	0.88	0.79	0.99	0.78
NT500	**0.9**	0.8	0.98	0.82	**0.91**	0.82	0.99	0.82
NT50	**0.9**	**0.82**	0.98	0.83	**0.91**	0.83	0.99	0.83
DeePhage	0.88	0.77	0.86	**0.91**	0.88	0.77	0.86	**0.91**
PhaTYP	0.77	0.53	0.74	0.79	0.77	0.53	0.74	0.79
2000 bp								
ProkBERT-mini	**0.97**	**0.93**	**1**	0.93	**0.97**	0.94	**1**	**0.94**
ProkBERT-mini-long	0.96	0.92	**1**	0.91	0.96	0.93	**1**	0.93
DNABERT-2	0.95	0.91	**1**	0.9	0.94	0.89	**1**	0.89
NT500	**0.97**	**0.93**	**1**	**0.94**	**0.97**	**0.95**	**1**	0.95
NT50	0.96	**0.93**	**1**	0.92	**0.97**	**0.95**	**1**	**0.94**
DeePhage	0.89	0.79	0.86	0.93	0.89	0.79	0.86	0.93
PhaTYP	0.83	0.67	0.93	0.72	0.83	0.67	0.93	0.72
10 000 bp								
ProkBERT-mini	0.96	0.93	**1**	0.93	**0.99**	**0.98**	**1**	**0.98**
ProkBERT-mini-long	0.98	0.95	**1**	0.95	0.98	0.95	**1**	0.95
DNABERT-2	0.95	0.91	**1**	0.9	0.94	0.89	**1**	0.88
NT500	0.98	0.95	**1**	0.95	0.96	0.93	**1**	0.93
NT50	0.98	0.95	**1**	0.95	0.96	0.93	**1**	0.93
DeePhage	**0.99**	**0.98**	**1**	**0.98**	**0.99**	**0.98**	**1**	**0.98**
PhaTYP	0.79	0.61	0.95	0.62	0.79	0.61	0.95	0.62

a Best performances in each segment are highlighted in bold. The metrics in the table are as follows: Acc.: accuracy, MCC: Matthews’ Correlation Coefficient, Sens.: sensitivity, and Spec.: specificity. Strict-Holdout refers to the setting where *Escherichia*-infecting phages and their relatives (quantified by sequence similarity) were excluded from the training data; Standard-Holdout shows where not.

Across all methods, accuracy improves as fragment length increases. At 500 bp, most models maintain high sensitivity (*geqq*0.98) but exhibit more modest specificity (0.80–0.83). When presented with 2000 bp fragments, both sensitivity and specificity routinely exceed 0.93. On 10 000 bp inputs, nearly all approaches achieve specificity and sensitivity above 0.95.

In this setting, the ProkBERT variants (mini and mini‐long) yield the highest accuracy (0.90–0.98) and MCC (0.82–0.95) at each fragment length. Notably, these models perform on par with or slightly ahead of larger transformer‐based architectures (e.g. DNABERT‐2 and NT), despite the latter having substantially more parameters. Convolutional‐neural‐network‐based DeePhage and the sentence‐embedding-based PhaTYP also exhibit strong accuracy on longer contigs but show somewhat lower specificity on 500 bp fragments, reflecting the inherent difficulty of very short inputs.

Adding *Escherichia*‐infecting phages to the training set produces only moderate enhancements for ProkBERT. Overall, these results indicate that ProkBERT PhaStyle remains effective even on novel, environmentally derived phage fragments.

#### 3.1.3 Out-of-sample prediction on the *Escherichia* (Guelin) collection

We evaluated each model on the Guelin collection of experimentally validated *Escherichia* phages, excluding any exact or closely related sequences from training (strict‐holdout) or permitting high‐similarity but nonidentical phages (standard‐holdout). Table 3 summarizes the results.

**Table 3.** Performance comparison of models on the *Escherichia* test set across various segment lengths.^a^

Model	Strict-Holdout (ANI ≧ 80 %)				Standard-Holdout			
	Acc.	MCC	Sens.	Spec.	Acc.	MCC	Sens.	Spec.
500 bp								
ProkBERT-mini	0.89	0.79	0.92	0.86	**0.93**	**0.86**	0.97	**0.89**
ProkBERT-mini-long	0.89	0.78	**0.94**	0.84	**0.93**	**0.86**	0.97	**0.89**
DNABERT-2	0.85	0.7	0.92	0.77	0.91	0.82	**0.98**	0.84
NT500	0.85	0.71	0.89	0.81	0.92	0.85	0.97	0.88
NT50	0.83	0.66	0.88	0.77	0.89	0.78	0.94	0.83
DeePhage	0.86	0.72	0.84	**0.88**	0.86	0.72	0.84	0.88
PhaTYP	**0.91**	**0.83**	**0.94**	**0.88**	0.91	0.83	0.94	0.88
2000 bp								
ProkBERT-mini	**0.96**	**0.92**	0.97	0.95	**0.97**	**0.94**	**0.98**	**0.96**
ProkBERT-mini-long	0.95	0.9	**0.98**	0.93	**0.97**	0.93	**0.98**	0.95
DNABERT-2	0.91	0.83	0.97	0.85	0.95	0.9	**0.98**	0.92
NT500	0.93	0.86	0.96	0.9	**0.97**	0.93	**0.98**	0.95
NT50	0.91	0.82	0.95	0.87	0.94	0.89	**0.98**	0.91
DeePhage	0.95	0.89	0.93	**0.96**	0.95	0.89	0.93	**0.96**
PhaTYP	0.92	0.84	0.97	0.86	0.92	0.84	0.97	0.86
10 000 bp								
ProkBERT-mini	**0.98**	**0.96**	**0.98**	0.98	**0.99**	**0.97**	**0.98**	0.99
ProkBERT-mini-long	**0.98**	**0.96**	**0.98**	0.98	0.98	**0.97**	**0.98**	0.99
DNABERT-2	0.96	0.91	**0.98**	0.93	0.98	0.95	**0.98**	0.97
NT500	0.97	0.94	0.97	0.97	0.98	0.96	**0.98**	0.99
NT50	0.95	0.9	0.96	0.94	0.97	0.94	**0.98**	0.97
DeePhage	**0.98**	**0.96**	0.96	**1**	0.98	0.96	0.96	**1**
PhaTYP	0.93	0.87	**0.98**	0.88	0.93	0.87	**0.98**	0.88

a Best performances in each segment are highlighted in bold. The metrics in the table are as follows: Acc.: accuracy, MCC: Matthews’ Correlation Coefficient, Sens.: sensitivity, and Spec.: specificity. Strict-Holdout refers to the setting where *Escherichia*-infecting phages and their relatives (quantified by sequence similarity) were excluded from the training data; Standard-Holdout shows where not.

Under strict holdout, classifying 500 bp fragments was most challenging: ProkBERT‐mini achieved 0.89 accuracy, while DNABERT‐2 and NT500 reached 0.85. As fragment length increased, all methods improved: at 2000 bp, ProkBERT‐mini rose to 0.96, with DNABERT‐2 at 0.91 and NT500 at 0.93. At 10 000 bp, ProkBERT‐mini and ProkBERT‐mini‐long reached 0.98 accuracy, closely followed by DeePhage (0.98) and NT500 (0.97). PhaTYP also benefited from longer fragments, however with lesser extent.

In the standard‐holdout setting—where high‐similarity phages inform the models—performance improved significantly. For example, ProkBERT‐mini attained 0.93 accuracy on 500 bp. All the gLM-based models outperformed the PhaTYP or DeePhage.

These results highlight three key points: (i) accuracy increases substantially with fragment length; (ii) ProkBERT variants generalize well to unseen *Escherichia* lineages, matching or exceeding larger models at all lengths; and (iii) when training includes high‐similarity phages, most methods converge to near‐perfect performance on longer contigs. Collectively, these findings underscore ProkBERT PhaStyle’s robustness and efficiency in predicting phage lifestyle directly from nucleotide sequences, even in challenging out‐of‐sample scenarios.

#### 3.1.4 Inference speed and running times

**Figure 2.**
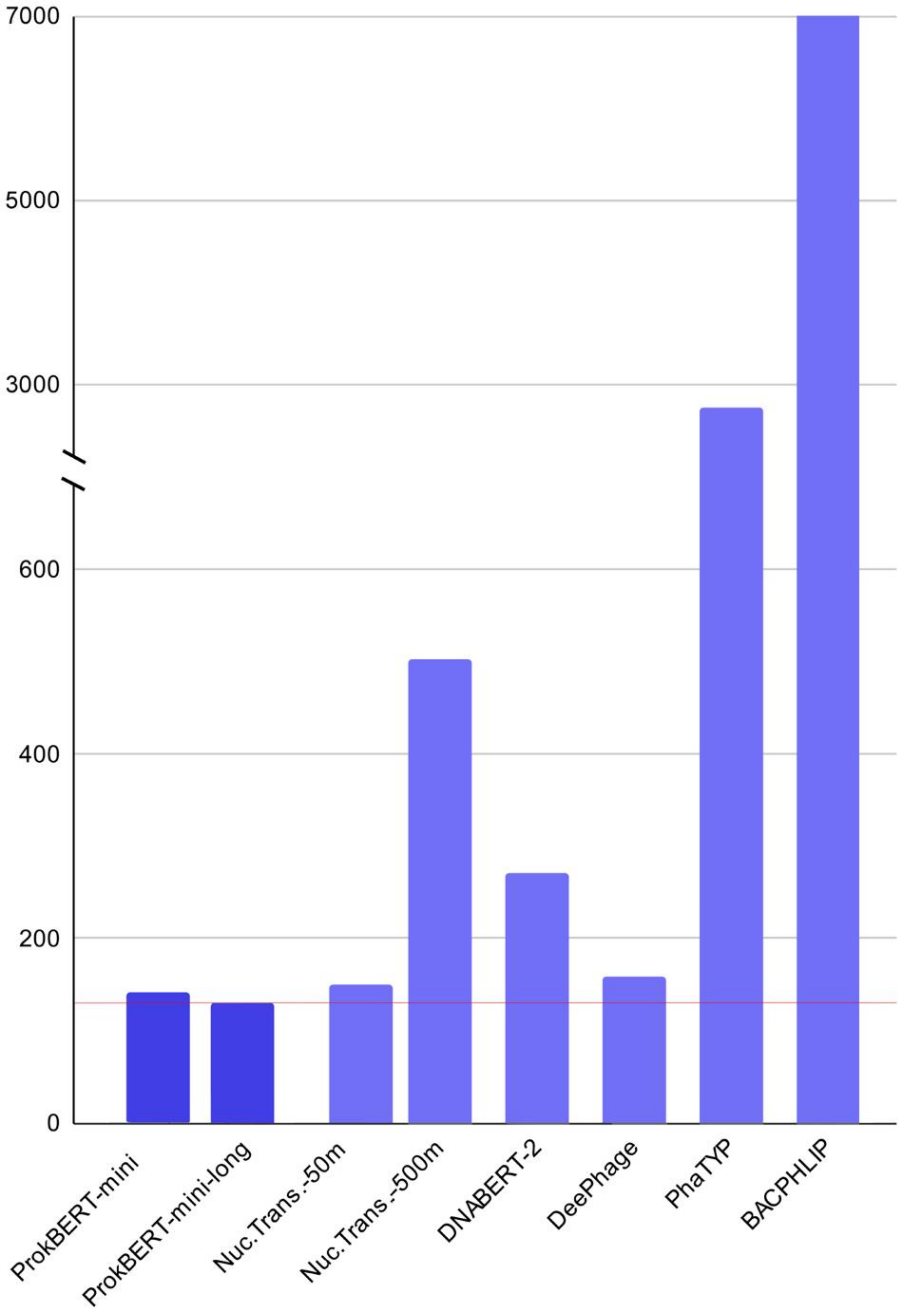
Execution times of various models for phage lifestyle prediction. The *y* axis represents the execution time in seconds, and the *x* axis lists the models. The line at 132s indicates the running time of the fastest model.

The computational performance of the models was assessed using a set of 1000 randomly selected sequences from the BACPHLIP dataset. The evaluations were conducted on identical hardware configurations (Fig. 2). Among the genomic LMs, ProkBERT-mini-long was the fastest with an execution time of 132 s and the highest inference speed of 0.52 MB/s. mini performed similarly, with a slightly longer execution time of 141 s, and inference speed of 0.49 MB/s.

As expected, larger transformer models such as DNABERT-2 (117 m parameters) and NT (500 m parameters) require 2×–3.8× more time to evaluate samples. Since these models apply different tokenization, the input size (number of tokens used in the representations) significantly influences the performance, i.e. the fewer tokens, the faster the prediction is.

DeePhage and PhaTYP displayed longer execution times and lower speeds compared to the genomic LMs. DeePhage executed in 159 s with a prediction rate of 0.43 MB/s. The database search-based approaches (PhaTYP and BACPHLIP) required significantly more time (2718 and 7125 s, respectively). (Table 5, available as supplementary data at *Bioinformatics Advances* online contains the data on execution time and inference speed.)

## 4 Discussion

In summary, the three datasets were selected so as to test the models’ performance and generalization capability. Interestingly, despite being placed at a disadvantage compared to PhaTYP, DeePhage that were trained on a broader dataset, the genomic LMs showed unexpectedly good prediction performance on all three datasets. One reason for this may be that, during the pretraining phase, they capture patterns that are useful for this task. Apparently, ProkBERT models consistently outperformed other models across various datasets and segment lengths. This is particularly notable since DNABERT-2 and NT models have much larger capacities (2–20 times more parameters) compared to ProkBERT. We believe this is due to the fact that ProkBERT was pretrained on a much larger microbial dataset and employs LCA tokenization, which provides more robust vectorization of nucleotide sequences.

### 4.1 Limitations and practical implications

Our proposed method, like existing phage lifestyle prediction tools, assumes input sequences are known to be viral. Both the genomic LMs and SOTA methods compared in this work are binary classifiers that require pre-identified phage sequences as input, labeling them as either virulent or temperate. Consequently, an upstream viral sequence identification step is necessary to distinguish phage sequences from non-phage sequences in metagenomic datasets.

To address this requirement, several viral identification tools can be integrated into the analysis pipeline, including VirSorter2 (Guo *et al.* 2021), VIBRANT (Kieft *et al.* 2020), DeepVirFinder (Ren *et al.* 2020), and ProkBERT (Ligeti *et al.* 2023), which is particularly effective on short fragments. For thorough reviews of these tools, see (Ho *et al.* 2023, Schackart *et al.* 2023, Wu *et al.* 2024). Additionally, metagenomic binning to group contigs into draft viral genomes before applying lifestyle prediction can enhance classification accuracy, and this preprocessing strategy is equally applicable to PhaStyle and all other existing tools.

The current models achieve reasonable accuracy on short contigs, but they are unable to capture the full genomic structure due to their architectural limitations. Future work will aim to incorporate such context to improve method reliability.

Another consideration is the computational resources required for model inference. Most of the models presented in this work, with the exception of BACPHLIP, perform best with GPU support due to the computational demands of deep learning models. While this may pose a challenge for some users, GPU resources are increasingly accessible. Cloud-based platforms like Google Colab offer free GPU access, and many institutions provide computational resources to researchers.

## 5 Conclusion

This study introduces and evaluates a novel approach for phage lifestyle prediction by applying pretrained genomic LMs and fine-tuning them for this specific task. The ability of these models, particularly ProkBERT, to directly process nucleotide sequences without the need for protein annotation or complex database searches offers various advantages, such as reducing biases and computational overhead.

ProkBERT, designed specifically for microbial classification tasks, consistently outperformed current computational approaches and other universal LMs, such as NT and DNABERT-2. The ProkBERT PhaStyle models proved to be simple, easy to train, and fast, demonstrating high efficiency and accuracy, even when classifying short sequence fragments. This underscores their applicability to metagenomic and virome datasets, where sequences are often fragmented.

These models offer a fast, alignment-free alternative to database-driven methods, with high accuracy and potential for improved generalization. Future research should aim to increase the interpretability of these models and explore their broader applications in microbial genomics.

## 6 Key points

We demonstrated that complex machine learning tasks such as phage lifestyle prediction can be solved directly from nucleotide sequences without the need for complex bioinformatics pipelines or manually curated protein annotations.

We showed that genomic LMs and the transfer learning paradigm offer a simple but efficient alternative to database search-based methods. They offer better generalization, while being faster and more accurate.

We introduced ProkBERT PhaStyle, which is compact and consistently outperformed alternative genomic LMs and alternative methods in various challenging scenarios, such as classifying short fragments, extremophile phage sequences, and unseen phage sequences.

## Supplementary Material

vbaf188_Supplementary_Data

## Data Availability

The datasets generated and analyzed for this study are available on Zenodo: 10.5281/zenodo.11482774. The various containerized versions of BACPHLIP, DNABERT-2, DeePhage, Nucleotide Transformers, and ProkBERT PhaStyle are available on Zenodo (Apptainer) and on Docker Hub https://hub.docker.com/repository/docker/obalasz/phage/general.

## Code availability

The code associated with this study is available on GitHub: https://github.com/nbrg-ppcu/PhaStyle. Additionally, datasets, models, and codes can be accessed at the following repositories: GitHub—https://github.com/nbrg-ppcu/PhaStyle and Hugging Face—https://huggingface.co/neuralbioinfo.
